# Compromised angiogenesis and vascular Integrity in impaired diabetic wound healing

**DOI:** 10.1371/journal.pone.0231962

**Published:** 2020-04-23

**Authors:** Uzoagu A. Okonkwo, Lin Chen, Da Ma, Veronica A. Haywood, May Barakat, Norifumi Urao, Luisa A. DiPietro

**Affiliations:** 1 Center for Wound Healing and Tissue Regeneration, University of Illinois at Chicago, Chicago, IL, United States of America; 2 Guangdong Provincial Key Laboratory of Stomatology, Stomatological Hospital, Guanghua School of Stomatology, SunYat-sen University, Guangzhou, Guangdong, China; 3 Department of Pharmacology, Upstate Medical University, Syracuse, NY, United States of America; Medical University Innsbruck, AUSTRIA

## Abstract

Vascular deficits are a fundamental contributing factor of diabetes-associated diseases. Although previous studies have demonstrated that the pro-angiogenic phase of wound healing is blunted in diabetes, a comprehensive understanding of the mechanisms that regulate skin revascularization and capillary stabilization in diabetic wounds is lacking. Using a mouse model of diabetic wound healing, we performed microCT analysis of the 3-dimensional architecture of the capillary bed. As compared to wild type, vessel surface area, branch junction number, total vessel length, and total branch number were significantly decreased in wounds of diabetic mice as compared to WT mice. Diabetic mouse wounds also had significantly increased capillary permeability and decreased pericyte coverage of capillaries. Diabetic wounds exhibited significant perturbations in the expression of factors that affect vascular regrowth, maturation and stability. Specifically, the expression of VEGF-A, Sprouty2, PEDF, LRP6, Thrombospondin 1, CXCL10, CXCR3, PDGFR-β, HB-EGF, EGFR, TGF-β1, Semaphorin3a, Neuropilin 1, angiopoietin 2, NG2, and RGS5 were down-regulated in diabetic wounds. Together, these studies provide novel information about the complexity of the perturbation of angiogenesis in diabetic wounds. Targeting factors responsible for wound resolution and vascular pruning, as well those that affect pericyte recruitment, maturation, and stability may have the potential to improve diabetic skin wound healing.

## Introduction

The increased incidence of Diabetes mellitus type 2 (DM2) is a global epidemic that affects both under-developed and developed countries including the United States. In 2014, the CDC reported the prevalence of diagnosed and undiagnosed DM2 in the USA as 30.3 million people, with an additional 84.1 million in a pre-diabetic state [1]. Both DM2 and pre-diabetes place individuals at an increased risk for comorbidities including cardiovascular disease, stroke, chronic kidney disease, and peripheral neuropathy [2]. One particularly notable complication of DM2 is the occurrence of skin wounds or ulcerations, often on the lower extremities [2]. Diabetic skin ulcerations present with associated destruction of multiple layers of dermal tissue, often including not only the epidermis and dermis, but in many cases, the subcutaneous tissue as well [3]. Fifteen percent of diabetic patients experience skin ulcerations on the lower extremities, most commonly occurring on the feet. The complication of diabetic foot ulcers (DFUs) leads 14–24% of affected patients to require lower extremity amputation, a high-risk and life-altering procedure with a five-year mortality rate approaching 50–59% [1–6]. Due to the significant contribution of non-healing wounds to diabetes-related mortality, a large body of research has focused on the pathophysiology of DFUs.

Normal wound healing consists of four key stages: hemostasis, inflammation, proliferation, and remodeling. In the proliferative stage of wound healing, angiogenesis involves the growth of blood vessels that are characterized by their immaturity, permeability, and redundancy [7]. This process is mediated by pro-angiogenic modulators, primarily vascular endothelial growth factor (VEGF) [8]. Once the healing wound bed is filled with immature granulation tissue and immature microvasculature, resolution factors including Sprouty2 (SPRY2) and pigment epithelium derived factor (PEDF) trigger vascular maturation and remodeling. Previously published data from our laboratory demonstrates that SPRY2 negatively regulates capillary growth while PEDF is responsible for much of the apoptosis driven vascular pruning that occurs during wound maturation [9, 10]. Wound maturation is further characterized by the stabilization of microvascular capillaries and the vascular basement membrane by a special population of mural cells called pericytes. Pericytes extend long cytoplasmic processes that wrap around endothelial cells, creating a close relationship between them. In recent years, the role of pericytes in vascular function has drawn much attention. Studies showed that pericytes not only interact with endothelial cells to regulate angiogenesis, but also interact with epithelial cells, fibroblasts, and leukocytes, all cell types that are involved in wound healing and tissue regeneration [11].

In the diabetic wound milieu, dysfunction affects all stages of wound healing, and diabetic wounds often fail to progress to stages of complete repair [12]. Prior studies have identified several factors as contributing to poor DFU healing, including microbial invasion, epithelial breakdown, and impaired immune function [13]. One underlying factor that impacts all diabetic ulcerations is impaired vascular circulation, which can lead to inadequate healing. Studies on vascular function in diabetic wound healing have, for the most part, focused on the impaired angiogenic phase that occurs in diabetic wounds [14]. Few studies have investigated the later phases of wound healing to determine whether alterations in maturity and vessel architecture might play role in the impaired healing of diabetes.

Using a diabetic mouse model, the study here examined how diabetes influences wound maturation, and in particular, the state of the capillary bed as healing resolves. Our study shows that blood vessels in the skin wounds of diabetic mice have less pericyte coverage and higher permeability. Wounds of diabetic mice exhibited significantly decreased expression of pro-angiogenic factors and factors responsible for pericyte recruitment and vessel maturation.

## Materials and methods

### Animals and wound preparation

Eight-week-old female C57Bl/6 wild type (WT) and db/db diabetic (BKS.Cg-Dock7m +/+ Leprdb/J) mice (The Jackson Laboratory, Bar Harbor, ME, USA) were utilized in this study. The total number of mice used in this study was 60 (29 C57Bl/6 WT and 31 db/db diabetic mice). For wound placement, mice were anesthetized with ketamine/xylazine (100/5 mg/kg) via intraperitoneal injection. After mice were completely anesthetized, as judged by the absence of a reaction to hind-paw squeezing, the dorsal skin was shaved, and hair was further removed with Nair depilatory cream (Church & Dwight, Ewing, NJ, USA) which was removed with gauze moistened with sterile deionized water. The skin was cleansed with 70% isopropyl alcohol. Two 8mm excisional full-thickness skin wounds were made on the dorsal skin of each mouse, equidistant from the vertebral column, using a biopsy punch (Acu Punch, Acuderm, Ft. Lauderdale, FL). Mastisol adhesive (Ferndale Laboratories, INC. Ferndale, MI, USA) was applied to the area surrounding the wound, and a Tegaderm bandage (3M, St. Paul, MN, USA) was placed. The excised skin removed during wounding was used as the uninjured baseline skin sample (day 0). After surgery, mice were placed under warming lamps and monitored until they recover from anesthesia, followed by daily observation until the end of the experiment. Mice were checked daily to ensure their well-being. Animals displaying distress such as difficulty breathing or wheezing, failure to thrive, abnormal grooming patterns, weight loss of more than 10%, bloody stool or diarrhea, or infection were euthanized prior to the end point of the study. At days 7, 10, 14, 18, and 22 post-wounding, mice were euthanized by CO2 inhalation and cervical dislocation, and the wound tissue was quickly harvested using a standard punch biopsy instrument. For reverse transcription polymerase chain reaction (RT-PCR) analysis, wound samples were stored in RNAlater (Sigma, St. Louis, MO, USA) at -20°C. For immuno-fluorescent staining, wound samples were embedded in Tissue-Plus OCT Compound (Fisher Scientific, Hampton, NH) and stored at -80°C. A total of 5 mice were used per experimental group. Mice were housed in groups of five at 22–24˚C on a 12 hours/12 hours light/dark cycle; water and food provided ad libitum. All animal protocols used in this experimental work were approved by the University of Illinois at Chicago Institutional Animal Care and Use Committee and all procedures followed the Guide for the Care and Use of Laboratory Animals (National Research Council).

### Wound closure measurements

Animals were anesthetized via isoflurane inhalation and photographed from a fixed distance, using a standard digital camera. Photographs were taken every other day. Images were analyzed using AxioVision software (ZEISS, Oberkochen, Germany). Wound size was calculated as: (measured wound area)/ (original wound area) ×100. The data from the two wounds on each mouse were averaged to produce a single value for each experimental animal.

### Model confirmation

Multiple previous studies demonstrated that db/db mice exhibit significantly delayed wound closure [15–17]. In the current study, the wounds of diabetic mice showed delayed wound closure at time points from day 3 through 19 post-wounding (S1A and S1B Fig). Complete wound closure occurred between days 11–13 in WT mice but did not occur until days 17–19 post-wounding in diabetic mice. These data verify that skin wounds in the db/db diabetic mouse model used here exhibited inherent deficits in re-epithelialization when compared to skin wounds in WT mice.

### Total RNA isolation, reverse transcription, and semi-quantitative RT-PCR

Excised skin wound samples were homogenized in TriZol (Invitrogen, San Diego, CA USA). DNA was digested and removed using Deoxyribonuclease I (Invitrogen), and RNA was subjected to reverse transcription using a High-Capacity cDNA Reverse Transcription Kit (ThermoFisher Scientific, Waltham, MA). Semi-quantitative mRNA expression analysis was performed using a SYBR Green polymerase chain reaction (PCR) mix and gene specific primers. Primers for selected mouse genes were designed using Online PrimerQuest Tool (IDT, Coralville, IA, USA) as listed in Table 1. Glyceraldehyde 3-phosphate dehydrogenase (*Gapdh*) was used as the standard housekeeping gene.

**Table 1:** Primer sequences for real time PCR.

Protein Name Abbreviation	Gene Name	Forward (5’——3’)	Reverse (5’——3’)
VEGF-A	*Vegfa*	CTGTGCAGGCTGCTGTAACG	GTTCCCGAAACCCTGAGGAG
SPRY2	*Spry2*	ACTGCTCCAATGACGATGAGGACA	CCTGGCACAATTTAAGGCAACCCT
PEDF	*Serpinf1*	TCGAAAGCAGCCCTGTGTT	AATCACCCGACTTCAGCAAGA
LRP6	*Lrp6*	CAGGCATGTAGCCCTTGGAG	ACTACAAGCCCTGCACTGCC
TSP1	*Thbs1*	TGGCCAGCGTTGCCA	TCTGCAGCACCCCCTGAA
CXCL10	*Cxcl10*	GTGGCATTCAAGGAGTACCTC	TGATGGCCTTCGATTCTGGATT
CXCR3	*Cxcr3*	CAGCCTGAACTTTGACAGAACCT	GCAGCCCCAGCAAGAAGA
PDGF-β	*Pdgfb*	ATCGCCGAGTGCAAGACGCG	AAGCACCATTGGCCGTCCGA
PDGFR-β	*Pdgfrb*	TCAACGACTCACCAGTGCTC	TTCAGAGGCAGGTAGGTGCT
HB-EGF	*Hbegf*	CAGGACTTGGAAGGGACAGA	GGCATTTGCAAGAGGGAGTA
EGFR	*Egfr*	TCTTCAAGGATGTGAAGT GTG	TGTACGCTTTCGAACAATGT
TGF-β1	*Tgfb1*	TTGCTTCAGCTCCACAGAGA	TGGTTGTAGAGGGCAAGGAC
ALK1	*Acvrl1*	GGCCTTTTGATGCTGTCG	ATGACCCCTGGCAGAATG
ALK5	*Acvrl5*	TGTGCACCATCTTCAAAAACA	ACCAAGGCCAGCTGACTG
SEMA3A	*Sema3a*	GGATGGGTCCTCATGCTCAC	TGGTGCTGCAAGTCAGAGCAG
NRP1	*Nrp1*	CTGGAGATCTGGGATGGATT	GGATAGAACGCCTGAAGAGG
ANG1	*Angpt1*	TGCAGCAACCAGCGCCGAAA	CAGGGCAGTTCCCGTCGTGT
ANG2	*Angpt2*	GCTTCGGGAGCCCTCTGGGA	CAGCGAATGCGCCTCGTTGC
TIE2	*Tie2*	GATTTTGGATTGTCCCGAGGTCAAG	CACCAATATCTGGGCAAATGATGG
α-SMA	*Acta2*	ACGGCCGCCTCCTCTTCCTC	GCCCAGCTTCGTCGTATTCC
NG2	*Cspg4*	AATGAGGACCTGCTACACGG	CATCTGTAGTCAACAGCCGC
Desmin	*Des*	TACACCTGCGAGATTGATGCC	GCGCAATGTTGTCCTGATAGC
RGS5	*Rgs5*	ATGTGTAAGGGACTGGCAGCTCTGCCGCAC	CTTGATTAGCTCCTTATAAAATTCAGAGCG
GAPDH	*Gapdh*	TCACCACCATGGAGAAGGC	GCTAAGCAGTTGGTGGTGCA

### Immunofluorescent histochemistry for CD31 and desmin

Wound samples were placed in Tissue-Plus OCT Compound (Fisher Scientific), frozen, and cryosectioned at a thickness of 10 μm per section. Frozen sections were air-dried for 10 min and rehydrated in PBS for 10 min, were then fixed in pre-cooled acetone for 30 seconds and washed in PBS three times for 3 min each time. Slides were blocked with 10% normal goat serum in PBS for 30 min at rt, followed by a wash with 1x PBS for 5 min. Sections were stained with rat anti-mouse CD31 (1:1600) (BD Pharmingen, San Diego, CA) and rabbit anti-human desmin (cross reacting with mouse) (1:2000) (Abcam, Cambridge, MA, USA) overnight at 4˚C. Slides were then washed 3x 5 min with 1 x PBS and incubated in Alexa Fluor 594 goat anti-rat and Alexa Fluor 488 goat anti-rabbit secondary antibodies (1:1000) (Invitrogen, San Diego, CA, USA) for 30 min at rt. Slides were then washed three times for 5 min each with 1 x PBS and mounted using 50% glycerol containing 4’,6-diamidino-2phenylindole (DAPI) for staining of cell nuclei. The stained and mounted sections were observed under a fluorescence microscope (Axioskop 40, ZEISS). Images of the wound edges and wound bed were recorded using a digital camera (AxioCam HRc, ZEISS) with the appropriate filters at 20× magnification and saved in JPEG format. Wound vascularity was quantified as the percentage of CD31 positive pixel areas over the total area using ImageJ as previously described [18]. To compare desmin and CD31 colocalization in day 10 and day 18 wounds, the level of capillaries without pericyte coverage, that is, those in which CD31 was not colocalized with desmin, was calculated as follows: [non-colocalized CD 31^+^ area]/[(total CD31^+^ area)]×100. At least two wound sections per animal and two wound images per section were analyzed and averaged to produce a single data point for each animal.

### Vascular permeability analysis

For vascular permeability analysis, at day 10 post-wounding, 200μl and 250μl of FITC/dextran (Sigma, 15mg/ml in PBS) were injected into the retro-orbital venous plexus of WT and db/db mice, respectively, 30 min before the animals were euthanized [9, 10]. The dosages were different to accommodate for the variation in blood volume between the WT and db/db mice [19]. Harvested wound samples were placed in OCT Compound, cryosectioned at a thickness of 10 μm, and stained with rat anti-mouse CD31 and Alexa Fluor 594 goat anti-rat secondary antibody as described above. The sections were mounted with 50% glycerol/PBS/DAPI as described above. Intra- and extra-vascular FITC/dextran positive pixel areas and the total defined areas in the wound bed were quantified using ImageJ. The percentage of intra- and extra-vascular FITC/dextran positive areas relative to the total wound area was calculated.

### Micro-computed tomography (microCT)

Detailed explanations of the methods of systemic vascular perfusion with X-ray contrast media (bismuth-gelatin), microCT scanning, image processing and resampling, three-dimensional reconstruction and segmentation, and three-dimensional analysis, can be found in our previous publication [20].

### Statistical analysis and data availability

The values of all data are expressed as mean ±SEM. Two-way ANOVA followed by Bonferroni’s post-tests (GraphPad Software, San Diego, CA, USA) and Wilcoxon Two Sample Exact Test (SAS Version 9.4) were used. Statistical significance was defined at a p value < 0.05.

All data is available at figshare.com. (https://doi.org/10.6084/m9.figshare.11553306.v1)

## Results

### 2-D and 3-D vascular architecture is significantly altered in diabetic wounds

**Fig 1:**
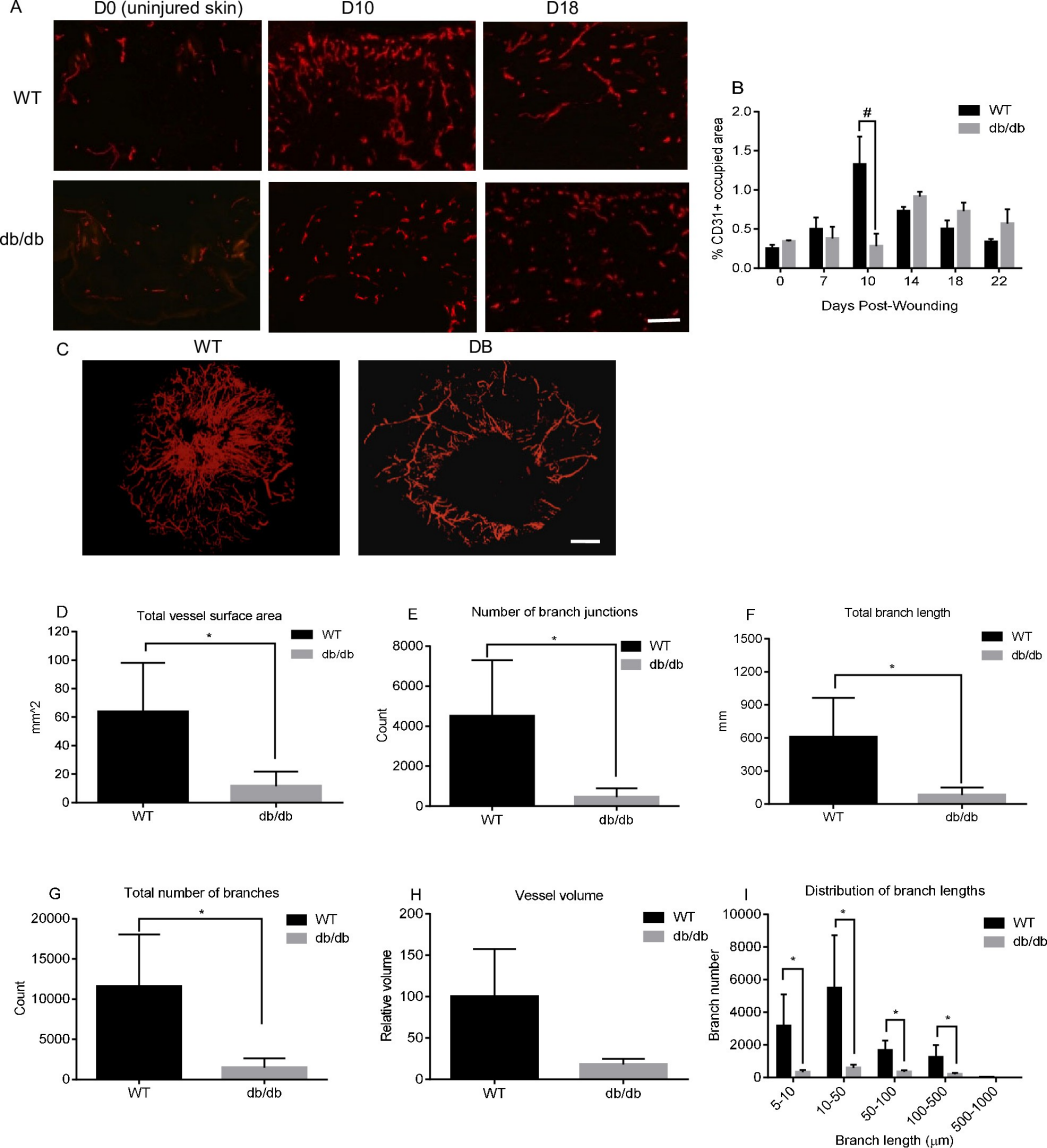
Diabetic wounds show altered 2-D and 3-D vascular architecture. Endothelial cells were identified by CD31 staining in baseline uninjured skin (day 0) and skin wounds at days 7, 10, 14, 18 and 22 post-wounding. A) Representative fluorescence photomicrographs of CD31 staining in the wounds. Scale bar = 100μm. B). Wound vascularity. The percent area of the wound edge and wound bed occupied by CD31-positive staining. Mean ±SD, n = 5. #p<0.01. C). MicroCT images of blood vessels in day 10 wounds of WT and diabetic mice. Scale bar = 1mm. D-I). Total vessel surface area, number of branch junctions, total branch length in (UNIT OF MEASUREMENT), total number of branches, total vessel volume, and the number of branches grouped by branch length, respectively, based on microCT image analysis. *p<0.05. n = 4 in WT group and 6 in diabetic group.

To compare capillary growth in the wounds of diabetic versus WT mice, a 2D assessment of capillary density was performed. Wound tissue sections from days 0, 7, 10, 14, 18, and 22 post-wounding were subjected to immunofluorescent staining for the endothelial cell marker CD31 followed by quantification of the percentage of CD31 positive area in the wounds (Fig 1A and 1B). For wounds in WT mice, the results showed that the level of capillaries increased over time, peaked at day 10 post-wounding, and returned to near baseline levels by day 22 (Fig 1A). In contrast, the pattern of angiogenesis in diabetic wounds showed both a delayed growth by several days and a decreased maximal level of capillary development. Most striking was the difference in capillary content at day 10 post-wounding, with WT wounds showing nearly 4 times as much capillary content as diabetic wounds at this time point (p< 0.01) (Fig 1A and 1B). This time point was of particular significance because previous studies in our lab showed that days 7–10 represent the peak of angiogenesis in normally healing wounds [9, 10, 21]. These results demonstrated a deficit in diabetic wound healing during the crucial switch from capillary growth to pruning and maturation.

Following the 2D analysis, wound angiogenesis was examined in 3D using microCT. This technique, described by us previously [20], was employed to examine the 3D vascular network in diabetic and control skin wounds at day 10 post-wounding (Fig 1C). The microCT analysis demonstrated multiple differences in the architecture of vessels in diabetic versus WT wounds. Vessel surface area (Fig 1D), branch junction number (Fig 1E), total vessel length (Fig 1F), and total branch number (Fig 1G) were significantly decreased db/db mice as compared to WT mice (p<0.05). A similar result was observed for the relative total vessel volume (Fig 1H), although no statistical significance was found (p = 0.06). We further separated the vessel branches into different groups according to their length (mm) and then counted the total number of branches in each group. The results revealed that the number of branches with lengths between 0.005–0.01mm, 0.01–0.05mm, 0.05–0.1mm, and 0.1–0.5mm in WT mice were significantly higher than those in diabetic mice (Fig 1I). Furthermore, video imaging of 3D vascular structures (S2 and S3 Figs) demonstrated that day 10 wounds in WT mice had significantly denser vascular network than in db/db mice. The microCT analysis showed that multiple alterations in wound capillary architecture occur in diabetic as compared to WT mice.

### Capillary permeability is increased and pericyte coverage is decreased in diabetic wounds

**Fig 2:**
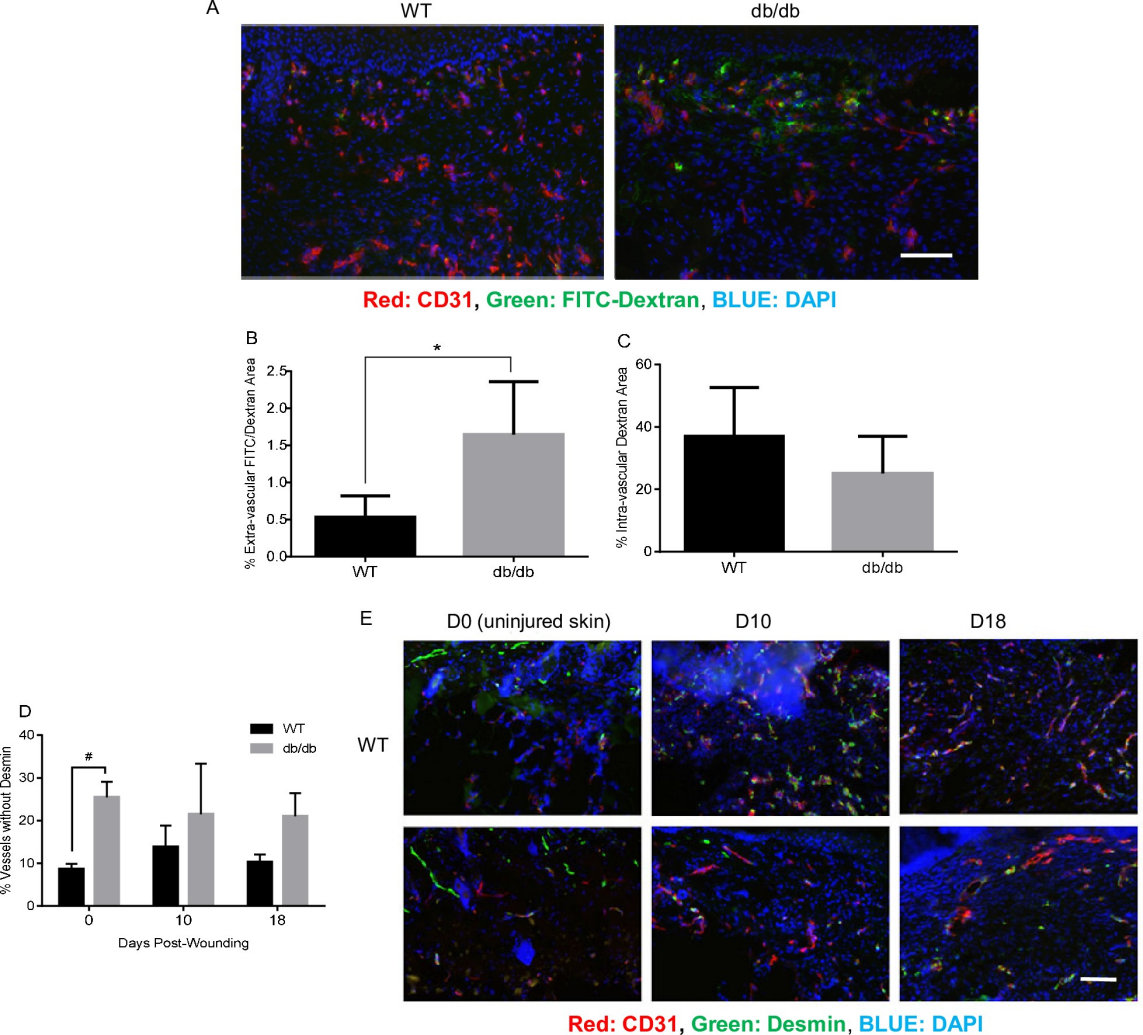
Diabetic wounds show increased amounts of vessel leakage and decreased pericyte coverage. A). Representative fluorescence photomicrographs of CD31- and DAPI-stained FITC-dextran perfused wounds. Scale bar = 100μm. B). The percent of extravascular FITC-dextran. *p>0.05, n = 5. C). The percent of intravascular FITC-dextran. n = 5. D). Comparative levels of endothelial cell—pericyte association, expressed as the percent of CD31+ vessels not colocalized with desmin, p<0.01, n = 5. E). Representative fluorescence photomicrographs of CD31- and desmin-stained uninjured skin (US), days 10 and 18 wounds. Scale bar = 100μm.

Next we investigated whether vascular permeability was altered in db/db versus WT mice skin wounds. Mice were injected with FITC-dextran conjugate, and the amount of extravascular FITC- dextran conjugate in the wound bed was examined as a marker of vessel permeability (Fig 2A and 2B). At day 10 post-wounding, diabetic skin wounds had a three times greater amount of extravascular FITC-dextran than wounds of WT mice (1.6% vs 0.5%, Fig 2A and 2B). Regarding intravascular FITC-dextran, diabetic wounds appeared to contain less intravascular FITC-dextran than WT wounds, although this difference was not statistically significant (Fig 2C). These results indicated that the vascular network in diabetic wounds exhibited greater permeability than that of WT controls.

As the capillary bed matures in wounds, vessels become covered with pericytes. To compare capillary maturity in wounds, the pericyte coverage of wound bed capillaries was compared in db/db and WT mice. Histologic sections were subjected to immunofluorescent staining for both desmin, a marker of pericytes, and CD31, a vascular marker. The number of capillaries with and without pericyte coverage was determined by counting the number of single positive (CD31^+^ only) and double positive (CD31^+^ and desmin^+^) cells in the wound bed (Fig 2D and 2E). Pericyte coverage was compared in uninjured skin (day 0) and at days 10 and 18 post-wounding, time points at which pericyte recruitment would be expected based on our wound closure data. Our results revealed that capillaries in both uninjured skin and wounds of diabetic mice exhibited a higher percentage of capillaries that were not associated with pericytes (i.e., not colocalized with desmin) when compared to WT (Fig 2D and 2E). The data suggested that when compared to WT, capillaries in diabetic skin have reduced pericyte coverage, with day 10 and day 18 wounds showing a trend toward reduced pericyte coverage.

### Regulators of angiogenesis and capillary maturity are differentially expressed in diabetic and WT mouse wounds

**Fig 3:**
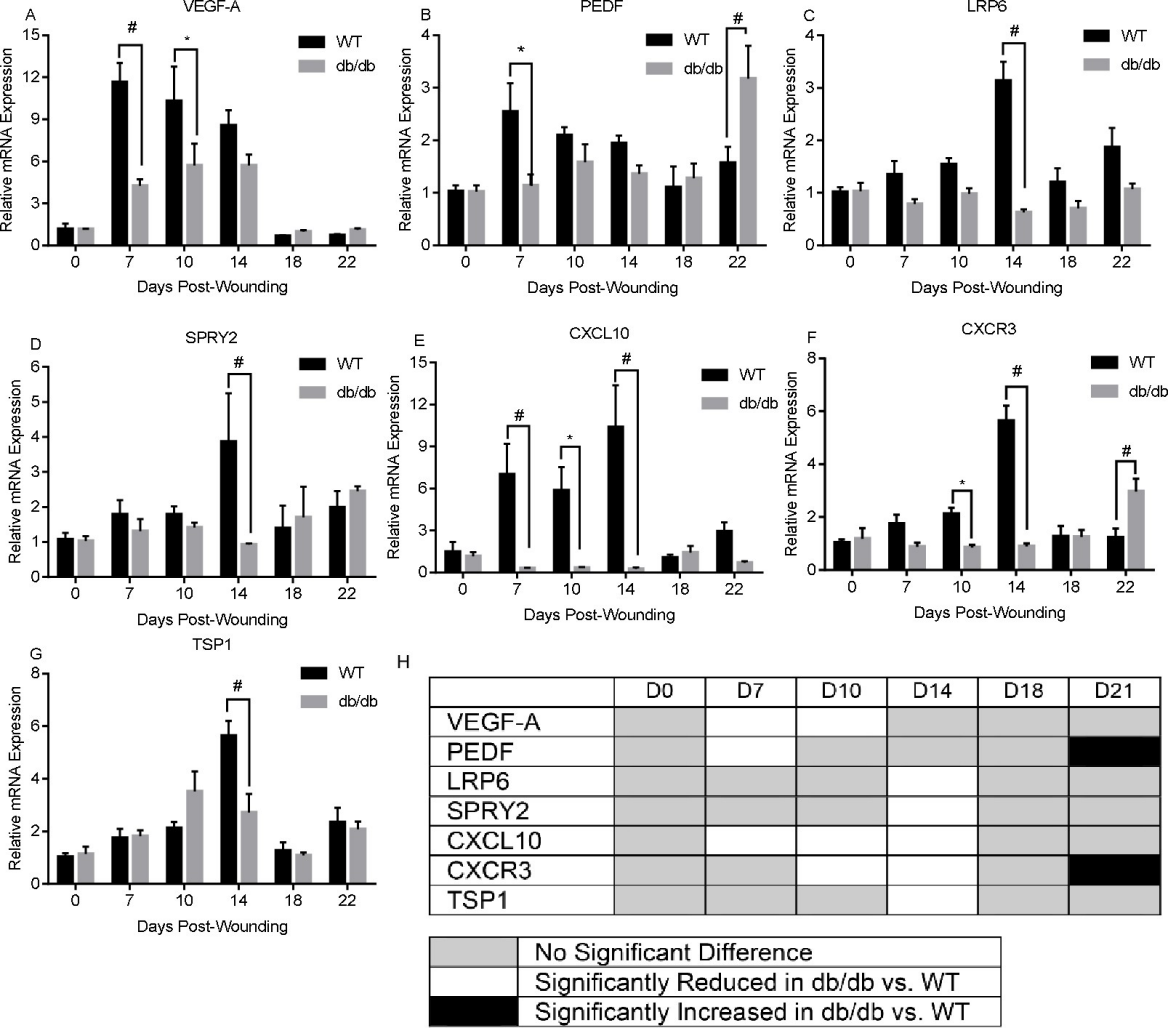
A-G) Semi-quantitative RT-PCR analysis of pro- and anti-angiogenic factors over the time course of wound healing. n = 5, *p<0.05, #p<0.01. H) Heatmap summarizing findings of A-G, showing genes with no difference (gray boxes)significant downregulation (white boxes) or upregulation (black boxes) over the time course of healing in db/db versus WT mice. In the figure, factors are identified using common protein name abbreviations; for gene symbols see Table 3.

**Fig 4:**
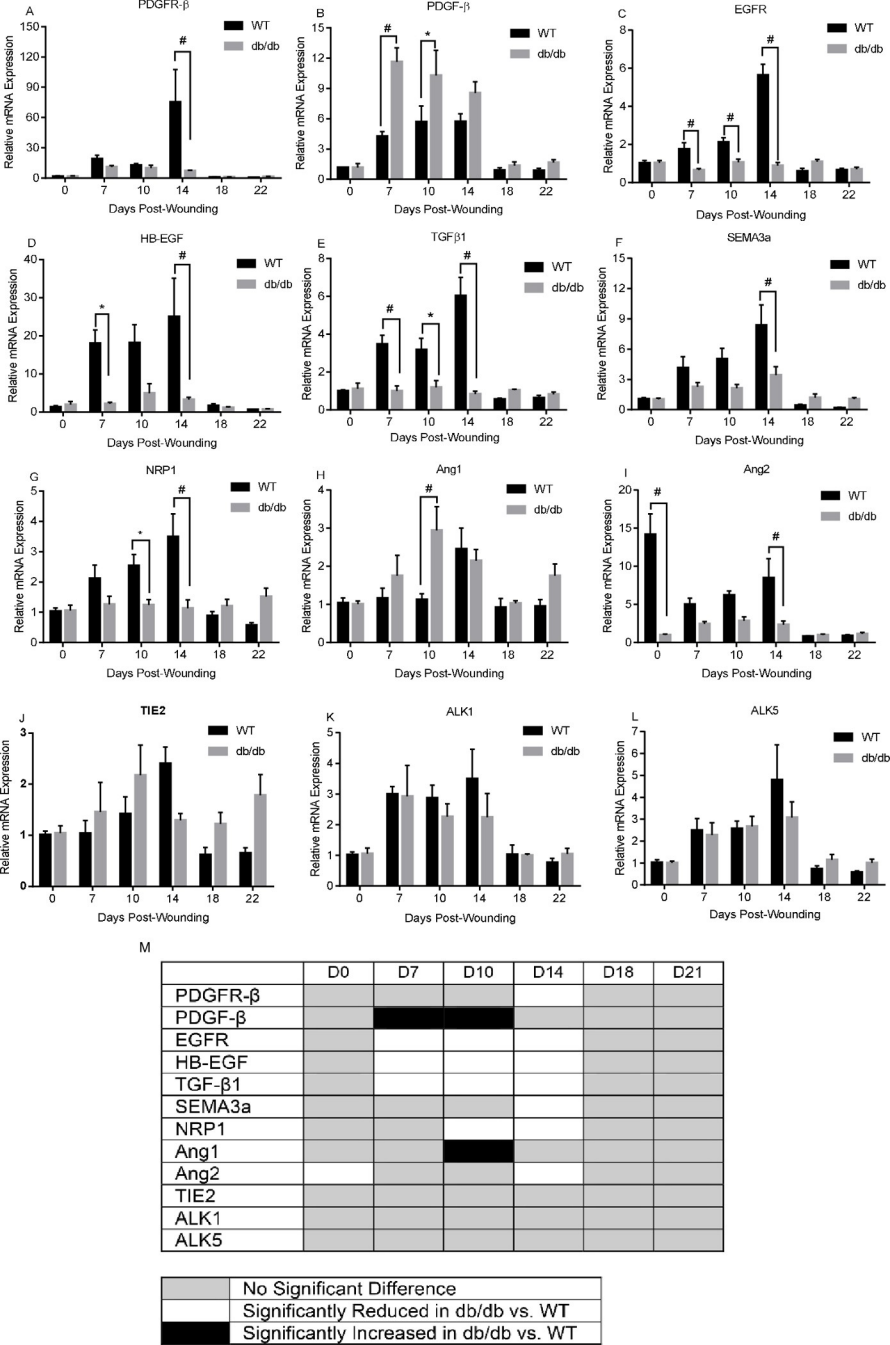
A-L) Semi-quantitative RT-PCR analysis of capillary pericyte recruitment and stabilization factors in wounds over the time course of wound healing. n = 5, *p>0.05, #p<0.01. M) Heatmap summarizing findings of A-L, showing genes with no difference (gray boxes) significant downregulation (white boxes) or upregulation (black boxes) over the time course of healing in db/db versus WT mice. In the figure, factors are identified using common protein name abbreviations; for gene symbols see Table 3.

**Fig 5:**
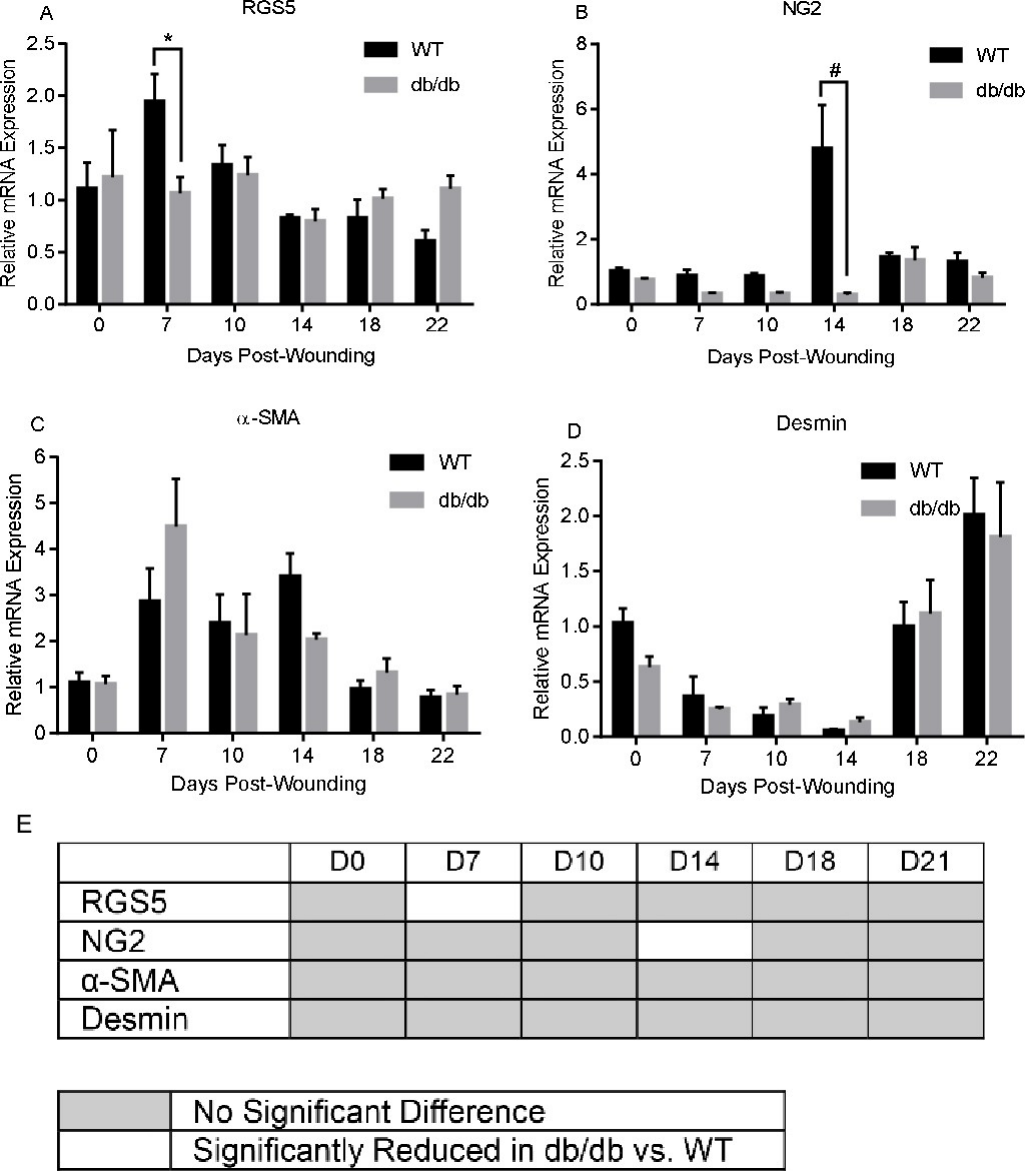
A-D) Semi-quantitative RT-PCR analysis of pericyte markers in mouse diabetic wounds over the time course of wound healing. n = 5, *p>0.05, #p<0.01. E) Heatmap summarizing findings of A-D, showing genes with no difference (gray boxes) significant downregulation (white boxes) or upregulation (black boxes) over the time course of healing in db/db versus WT mice. In the figure, factors are identified using common protein name abbreviations; for gene symbols see Table 3.

To determine if alterations in the expression of angiogenic regulators might underlie the observed changes in wound angiogenesis in db/db versus WT mice, we used semi-quantitative RT-PCR to determine the relative mRNA levels for a panel of 1) pro- and anti-angiogenic factors (Fig 3), 2) factors that affect capillary recruitment, maturation, and stability (Fig 4), and 3) markers for pericytes (Fig 5) (Table 2).

**Table 2:** Proangiogenic, capillary pruning/maturation, and pericyte-associated factors and receptors.

Functional Category	Protein Name Abbreviation	References
Proangiogenic	VEGF-A	[8]
Capillary Pruning/Maturation	SPRY2	[33,36–38]
	PEDF/LRP6	
	TSP1	
	CXCL10/CXCR3	
Pericyte Recruitment/Stabilization	PDGF-β/PDGFR-β	[22]
	HB-EGF/EGFR	[23]
	TGF-β1/ALK1, ALK5	[24]
	SEMA3a/NRP1	[25]
	Ang1, Ang2/Tie2	[26,27]
Pericyte Markers	NG2	[28,29]
	α-SMA	
	RGS5	
	Desmin	

In the assessment of pro- and anti-angiogenic factors, VEGF-A, a factor known to be the primary pro-angiogenic mediator in wounds, was found to be significantly decreased in wounds of db/db mice compared to WT mice at both day 7 and day 10 post-wounding (Fig 3A and Table 3). We then analyzed several wound resolution factors that are known to influence capillary regression and maturation (Table 2). PEDF, LRP6 (a PEDF receptor), Sprouty 2 (SPRY2), CXCL10, CXCR3, and TSP1 were all significantly down-regulated in db/db versus WT wounds at days 7, 10 and/or 14 (Fig 3B–3G and Table 3). Although the general pattern for resolution factors was decreased expression under the condition of diabetes, both PEDF and CXCR3 showed significantly higher expression in db/db versus WT wounds at day 22 (Fig 3B and 3F and Table 3). Overall, the results suggest that both capillary growth and pruning are perturbed in diabetic wounds.

**Table 3:** Summary of gene expression changes in diabetic wounds as compared to normal wounds.

Gene Name	Protein Name (Abbreviation)	Change in Diabetic Wound
Vegfa	Vascular endothelial growth factor A (VEGF-A)	↓
Spry2	Sprouty 2 (SPRY2)	↓
Serpinf1/Lrp6	Pigment epithelium derived factor (PEDF)/ Low-density lipoprotein receptor-related protein 6 (LRP6)	↓/↓
Thbs1	Thrombospondin 1 (TSP1)	↓
Cxcl10/Cxcr3	C-X-C motif chemokine 10 (CXCL10, IP-10)	↓/↓
	CXC chemokine receptor 3 (CXCR3)	
Pdgfb/Pdgfrb	Platelet derived growth factor sub unit B (PDGF-B)/ Platelet derived growth factor receptor β chain (PDGFR-β)	↑/↓
Hbegf/Egfr	Heparin-binding EGF-like growth factor (HB-EGF)	↓/↓
	Epidermal growth factor receptor (EGFR)	
Tgfb1/ Acvrl1/ Acvrl5	Transforming growth factor β1 (TGFβ1)/Actin receptor-like kinase 1, 5 (ALK 1, 5)	↓/↔/ ↔
Sema3a/Nrp1	Semaphorin3a /Neuropilin1	↓/↓
Angpt1/ Angpt2/Tie2	Angiopoietin 1, 2 (Ang1, 2) /Tyrosine kinase with immunoglobulin-like and EGF-like domains (Tie2)	↑/↓/ ↔
Acta2	α-smooth muscle actin (αSMA)	↔
Cspg4	Neuron-glial antigen 2 (NG2)	↓
Rgs5	Regulator of G-protein signaling 5 (RGS5)	↓
Des	Desmin	↔

We next investigated factors that are involved in pericyte recruitment and stabilization (Table 2). Most of these factors, including PDGFR-β [22], EGFR, HB-EGF [23], TGFβ1 [24], SEMA3a, NRP1 [25], and Ang2 [26, 27], showed significantly decreased expression in diabetic wounds at particular time points between days 7 and 14 (Fig 4A, 4C–4G and 4I and Table 3). Conversely, PDGF-B and Ang1 showed significantly increased expression at days 7 and/or 10 in diabetic wounds as compared to WT (Fig 4B and 4H and Table 3). Tie2 and two TGFβ receptors, ALK1 and ALK5, did not show any significant difference in expression levels between diabetic and WT wounds at any time point that was examined (Fig 4J–4L and Table 3). Thus, consistent with the decreased levels of pericytes seen in diabetes (Fig 2), the production of factors involved in pericyte recruitment is perturbed in diabetic wounds.

Finally we assessed a group of factors that are known to be pericyte markers [28, 29] (Table 2). Despite the histologic evidence of altered levels of pericytes in diabetes, few pericyte markers showed differential expression in db/db and WT wounds. Only regulator of G-protein signaling 5 (RGS5) and NG2 exhibited significantly higher expression in WT than db/db mice at day 7 and 14 respectively (Fig 5 and Table 3). This result may stem from the fact that pericytes comprise a very small proportion of cells in skin, making differential analysis by RT-PCR challenging.

The results of the semi-quantitative RT-PCR analysis of the expression of factors that modulate angiogenesis are summarized in Table 3. Diabetic wounds displayed significant changes in expression of factors associated with proper recruitment of endothelial cells, as well as factors responsible for the maturation of blood vessels.

## Discussion

Wound healing is a dynamic process characterized by hemostasis, inflammation, proliferation, and remodeling. In diabetes, dysfunction in all stages of wound healing impairs the ability of injured tissues to progress to healing and regeneration. Particularly in DM2, vascular complications can lead to systemic effects throughout the body. These vascular deficits can take the form of cardiovascular disease, which ultimately leads to peripheral vascular disease, a condition that weakens the peripheral vessels and impairs their proper function [30]. This condition renders capillary endothelial cells prone to apoptosis and detachment in the diabetic state [31], and microangiopathies are common in diabetic skin [32]. The findings of the current study suggest that the decreased amount of functional endothelial cells present in the diabetic skin leads to a decreased amount of nascent microvasculature in the wound. In diabetic wounds, the deficit in angiogenesis has been thought to be primarily due to decreased expression of pro-angiogenic factors such as VEGF [12]. The studies here utilized a detailed microCT analysis to demonstrate that angiogenesis in diabetic wounds is highly dysfunctional in aspects beyond capillary growth. Our findings do corroborate prior studies that show that capillary content is decreased in the proangiogenic phase of repair in diabetic wounds. The microCT data extends this understanding to show that the capillaries in diabetic wounds are highly tortuous, a feature suggestive of poor perfusion. Moreover, pruning and refinement of the capillary bed, a process essential to adequate wound resolution and tissue restoration, appears to be impaired in the condition of diabetes. This new finding, that capillary maturation in wounds is impaired in diabetes, is supported by the finding that diabetic wounds exhibit altered levels of multiple factors that are required to prune and refine wound vasculature [33].

The current study shows that pruning of microvessels is impaired in diabetic wounds, and that diabetic wounds exhibit a delay in capillary maturation. Once endothelial cells produce immature microvessels, maturation requires signals from pericytes, which promote the integrity and stability of vessel beds. Pericytes, which are mural cells that envelope blood vessels, stabilize capillaries and protect them from anti-angiogenic factors and other stimuli [34, 35]. As further discussed below, the data here demonstrates that pericyte coverage is delayed in diabetic wounds. Overall, then, the changes in wound angiogenesis in diabetes are numerous, and are reflective of a large number of changes in the expression of the factors that regulate this process.

The current investigation provides significant new information about the differential expression patterns and possible impact of numerous modulators of vascular growth, pruning, and pericyte recruitment in diabetic wound healing. Many of these factors have never been characterized in wounds, skin, or in the context of DM. In this study, the previously well-described loss of VEGF activity in diabetic wounds was found to be accompanied by significant changes in expression levels of multiple factors that function during wound remodeling. Significantly lower levels of CXCR3 and its ligand, CXCL10, both of which are well known to be important to wound resolution [36], were observed in diabetic wounds. The expression of SPRY2 [37], TSP1 [38], and PEDF [9, 10], all factors that are known to promote vascular pruning in wounds, was also significantly decreased in wounds from db/db mice. Interestingly TSP1 may play a dual role in wounds, as it can affect not only local capillary regression, but also has been described to have a positive effect on the activity of vascular precursor cells. In this case, a decrease in TSP1 might suppress the important activity of vascular precursor cells in the wound, and might also inhibit the maturation of the wound capillary bed at later stages. [39]. SPRY2 is also multi-functional, and may down-regulate vascularity and/or vascular branching during wound angiogenesis [37]. A loss of both of these SPRY2 functions is in concert with the microCT finding of tortuous vessels in the diabetic wounds and delayed pruning. Similar to TSP1 and SPRY2, diminished levels of LRP6 (the PEDF receptor) were observed in wounds from db/db mice. Together, these data suggest that the capillary refinement phase of wound healing, a phase essential to the development of a well-perfused vascular bed, is impaired in diabetic wound healing.

The current studies demonstrate that the recruitment of pericytes to the new capillary bed is delayed in the context of diabetic wounds. Pericytes are thought to have pluripotent, stem cell-like properties that assist in the regulation of capillary blood flow and vessel stabilization [40, 41]. Pericytes envelope the outside surface of blood vessels and work in concert with endothelial cells that form the inner lining via paracrine communication [28, 42, 43]. Decreases in pericyte coverage of capillaries can cause extravascular leakage and edema of the vasculature [44]. The importance of pericytes in wound healing has been demonstrated in only a few prior studies. Of note, in a mouse model, imantinib (a tyrosine kinase inhibitor) treatment led to delayed wound healing and decreased levels of pericytes in dermal wound tissue [45]. While many markers of pericytes have been described, desmin, an intracellular class III intermediary filament protein found in skeletal, cardiac, and smooth muscle, is a useful tool for histologic analysis. Desmin has been shown to be closely associated with newly formed blood vessels making it a superior choice for immunofluorescent histochemistry analysis of pericyte coverage [28]. Using desmin as a marker of pericytes, we determined the extent of pericyte coverage on wound capillaries. The data shows that diabetic wounds exhibit fewer capillaries with concurrent desmin expression or, expressed another way, more vessels without desmin coexpression, throughout the time course of wound healing. These data suggest that the completion of pericyte coverage is significantly delayed in diabetic wounds. Moreover, uninjured skin of db/db mice, when compared to uninjured skin of WT mice, had significantly reduced pericyte coverage, with up to 30% of vessels appearing pericyte negative. This finding indicates that diabetic wounds probably never fully return to normal pericyte coverage, even if complete wound closure is achieved. The determination of whether this baseline vascular abnormality of capillaries in diabetic skin may predispose the tissue to breakdown and subsequent ulceration will be an important area of future investigation.

The reduced pericyte recruitment in diabetic wounds seems to have a multifactorial mechanism. Nearly all of the pericyte recruitment factors and receptors that were examined, including HB-EGF, EGFR, TGFβ1, SEMA3a, and NRP1, exhibited decreased expression in wounds of db/db mice compared to WT mice. Among the pericyte recruitment factors, only PDGF exhibited increased mRNA in the wounds of db/db versus WT mice. However, the increase in PDGF is juxtaposed with a decrease in the PDGF receptor (PDGFRβ). Therefore, although PDGF was transiently elevated, a decrease in receptors could reduce the effect of PDGF, a situation that has been suggested by other studies [46]. Interestingly, prior studies have shown that PDGF-BB supplementation does not improve diabetic wound healing in mice, perhaps due to decreased receptor status [47]. The findings for PDGF are in contrast to the TGFβ –ALK1/5 results. While TGFβ was decreased, ALK1 and ALK 5 did not exhibit any change in diabetic wounds. Therefore, TGFβ receptors appear to be present in sufficient quantities for appropriate activity. In thinking about diabetic wound treatments, a consideration of receptor levels seems critical. In the case of therapeutics that involve growth factors, effective treatment would require that the tissue exhibits adequate receptor expression.

In addition to pericyte recruitment factors, another group of factors critical to vascular stabilization, Tie2 and its ligands Ang1 and Ang2, were examined. Ang1 expression levels were increased at d10, while Ang2 expression levels were decreased in wounds of db/db mice. Although the literature on the effect of diabetes on the Tie2-Ang1/2 axis is somewhat conflicting, studies in human diabetic subjects have suggested that both Ang1 and Ang2 are regulated by diabetes, and more specifically, that their production changes in response to glycemic control [48]. Whether this control mechanism is in play in the diabetic wound will require more study. Overall, though. Our results suggests that in diabetic wounds, the Tie1 receptor remains engaged by the capillary destabilizing factor Ang1 for a longer period of time than in WT mice. Diabetic wounds may experience a relatively delayed return to Ang2 engagement and capillary maturation.

Our findings suggest that additional investigations using gain or loss of function assays are needed to determine the mechanisms underlying the changes seen in wound angiogenesis in diabetes. For example, our previous studies show that loss of PEDF delays vessel regression and collagen maturation in wounds [9, 10], whereas the addition of the factor reduced angiogenesis but promoted vessel integrity. While it seems counterintuitive to apply an anti-angiogenic factor to diabetic wounds, such an approach might improve healing outcomes if the timing and dose was correct.

In conclusion, diabetic skin wounds exhibit significantly less neovascularization and pro-angiogenic factor expression after injury, along with lower expression of factors responsible for vessel maturation and pericyte recruitment. Targeting these factors has the potential to improve diabetic skin wound healing, and future investigation into their individual functions may reveal potential novel therapeutics, modulators, or preventative methods of treatment for the impaired healing phenotype in diabetic wounds.

## Supporting information

S1 Fig Wound closure rates in db/db versus WT mice. Two 8mm punch wounds were made on the dorsal skin of WT and db/db mice. Wounds were photographed every other day. A). Representative photographs of the time course of wound healing. Scale bar = 4mm. B). The percent closure determined using image analysis of standardized photographs. n = 5 for each mouse group. *p<0.05, #p<0.01.
(PDF)

S2 Fig (MOV)

S3 Fig (MOV)
