# Supplementary material for: Compromised angiogenesis and vascular Integrity in impaired diabetic wound healing
Source: PLoS One. 2020 Apr 23;15(4):e0231962. doi: 10.1371/journal.pone.0231962 (PMC7179900; doi:10.1371/journal.pone.0231962)
Supplement: S1 Fig — Two 8mm punch wounds were made on the dorsal skin of WT and db/db mice. Wounds were photographed every other day. A). Representative photographs of the time course of wound healing. Scale bar = 4mm. B). The percent closure determined using image analysis of standardized photographs. n = 5 for each mouse group. *p<0.05, #p<0.01. (PDF) [file pone.0231962.s001.pdf]

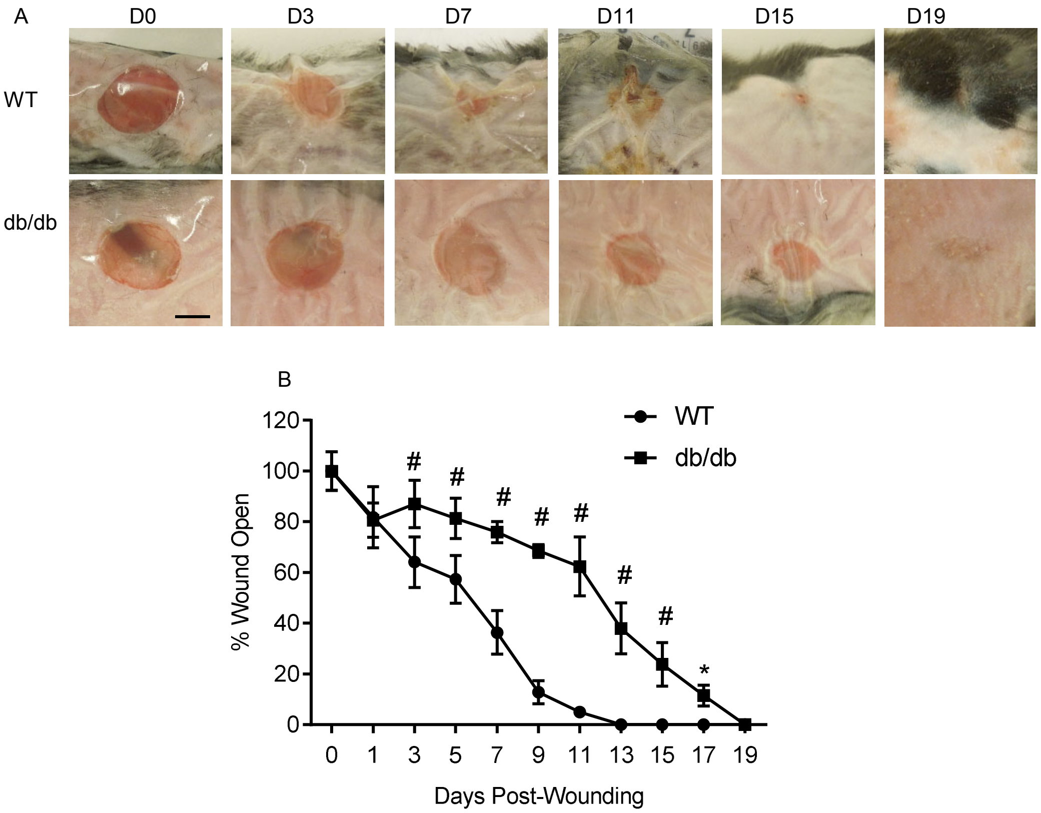

Supplemental Figure 1. Wound closure rates in db/db versus WT mice. Two 8mm punch wounds were made on the dorsal skin of WT and db/db mice. Wounds were photographed every other day. A). Representative photographs of the time course of wound healing. Scale bar=4mm. B). The percent closure determined using image analysis of standardized photographs. n=5 for each mouse group. *p<0.05, #p<0.01.
